# The Future of Minimally Invasive GI and Capsule Diagnostics (REFLECT), October 2024

**DOI:** 10.3390/diagnostics15070859

**Published:** 2025-03-27

**Authors:** Lea Østergaard Hansen, Alexandra Agache, Anastasios Koulaouzidis

**Affiliations:** 1 Department of Surgery, Odense University Hospital, 5700 Svendborg, Denmark; alexandra.agache@rsyd.dk (A.A.); anastasios.koulaouzidis@rsyd.dk (A.K.); 2 Department of Clinical Research, University of Southern Denmark, 5230 Odense, Denmark; 3 Department 10 General Surgery, University of Medicine and Pharmacy “Carol Davila”, 050474 Bucharest, Romania

**Keywords:** conference, gastrointestinal, minimally invasive diagnostics

## Abstract

The fifth annual REFLECT (The futuRE oF MinimalLy InvasivE GI and Capsule diagnosTics) symposium, held in October 2024 in Nyborg, Denmark, focused on advancements in minimally invasive gastrointestinal (GI) diagnostics, particularly capsule endoscopy (CE) technologies. Key discussions included clinical updates, innovations in hardware and software, and the growing role of colon CE (CCE) in colorectal cancer screening. The event provided a platform for clinicians, engineers, industry representatives, and scientists to exchange knowledge and present the latest advancements in the field. Discussions covered clinical studies, future research protocols, and technological innovations, with also a notable focus on commercial solutions and expansion of the implementation of capsule endoscopy. The symposium also highlighted the significance of predictive models for patient selection and developments in panenteric CE. Innovative technologies presented included robotics for drug delivery and magnetic endoscopic guidance systems. AI advancements were discussed for their potential to reduce diagnostic fatigue and standardize image interpretation, but ethical concerns and the need for transparent algorithms remain. The importance of multidisciplinary collaboration was emphasized to bridge innovation and clinical practice. Home-based CCE delivery emerged as a promising model, despite mixed results from environmental impact assessments. Overall, REFLECT 2024 reinforced the clinical utility and challenges of capsule-based diagnostics, advocating for ongoing interdisciplinary research to support safe and effective integration into healthcare systems.

## 1. Introduction

In October 2024, the fifth annual REFLECT symposium took place in Nyborg, Denmark. The Future of Minimally Invasive Gastrointestinal and Capsule Diagnostics (REFLECT) again brought together leading researchers and experts from the field—clinicians, engineers, industry representatives, and scientists—to share knowledge and present the latest advancements in gastrointestinal (GI) diagnostics.

Over the last few years, a multidisciplinary group of experts have met to provide a place for exchanging knowledge and experience, while strengthening professional networks among the leading experts in the field. Each year, the REFLECT program includes the latest insights from clinical studies and discussions on future study protocols and ideas. Additionally, the program consistently incorporates the latest developments in both hardware and software. This year, a particular notice was made to discuss commercial solutions and expand the implementation of CCE.

The conference was initiated with a statement acknowledging that the global population has reached 8 billion, most of whom will eventually be screened for colorectal cancer, a positive diagnostic for 9% of them. Improving and perfecting the diagnostic process is in the interest of many million people, highlighting the importance of this symposium. This paper will summarize the symposium’s discussions, which included 25 lectures across five sessions, each concluding with a comprehensive panel discussion.

## 2. Clinical Session I—Colon Capsule Endoscopy and Capsule Panendoscopy

The first session of REFLECT 2024 highlighted new information and insights from some of the largest clinical trials that have been performed on the use of colon capsule endoscopy (CCE) in clinical settings [1,2,3,4,5,6]. A study from the UK, which included data from 4878 CCE investigations, 5025 colonoscopies, and 466 CT colonographies performed from 2021 through 2024, reported higher polyp detection rates for both smaller and larger polyps with CCE than colonoscopy and CT-colonography [5]. Previous studies have also found CCE to be comparable to colonoscopy when it comes to adenoma detection and cancer detection [1,7].

The UK study, furthermore, reported that completion rates and the bowel preparation of CCE were lower than those for colonoscopy. The total of patients having a complete and adequate investigation was only 63%, while for patients undergoing colonoscopy or CT-colonography, it was 88% [5]. The interim analysis of a large Danish CCE study found the completion rate to be 67.9%, with 80.3% of the investigations being conclusive [6].

When comparing CCE and colonoscopy, it is essential to evaluate all aspects of both modalities. Colonoscopy is an invasive test, even though the risk of complications is low, the possible complications may be of great impact for patient bleeding, perforation, and infections [8,9]. However, colonoscopy remains the gold standard for the investigation of the colon, mainly due to the therapeutics aspects, even though the main demand is for diagnostics [10]. As the risk of complications is elevated with age and for patients with inflammatory bowel disease, it seems reasonable to investigate the value of using CCE as a diagnostic modality for these patient group [9].

One of CCE’s primary benefits is its accessibility. Even before the COVID-19 pandemic, waiting times for colonoscopies posed challenges in Scotland, England, and Ireland—an intensified issue [11]. The availability of colon capsule endoscopy can help reduce waiting times and categorize urgent patients to be investigated first. By incorporating colon capsule endoscopy alongside colonoscopy, healthcare systems could alleviate resource strains [12]. The trials on CCE generally report high sensitivity, both per person and per polyp, and it generally does not miss cancer, making CCE a good tool for investigating the colon [1,5]. As discussed at previous year’s REFLECT 2023, the main drawback is that CCE’s cost-effectiveness remains limited; however, CCE is still limited by the lack of intervention, when compared to colonoscopy, resulting in reinvestigations for patients with incomplete investigations or significant pathology. Which results in a limitation of the cost-effectiveness of CCE, primarily due to the cost of the capsules and the need for additional follow-up procedures [13,14,15]. The reinvestigations are due to incomplete capsule investigation or pathology requiring further intervention [16]. Due to the lack of evidence in the area, the real cost-effectiveness of CCE is still to be determined and agreed upon and needs to be fully evaluated before deciding on the use of CCE in clinical settings.

One way to improve cost-effectiveness is by developing a predictor model for patients with a low risk of incomplete investigation and no urgent pathology. For instance, studies on the ScotCap population indicated that younger age was associated with higher odds of having a successful CCE test [17]. The study also showed a demographic difference in acceptable pre-procedural cleansing [17]. This subject is of great interest because of the need to find the optimal group for CCE investigation—the group with a complete investigation without urgent pathology. On the other side, if the model can predict which patients will have no urgent pathology, an important question arises: will the patients even need testing?

Another subject drawn to attention in this session was the panenteric CE (PCE), which is becoming increasingly relevant [18]. Studies on the feasibility of PCE indicate that it is an effective and safe diagnostic method, with the advantage of investigating the entire GI tract in a single procedure [18,19].

Future studies and trials are in the pipeline; one of them is the ColoCap trial, which also aims to investigate the diagnostic accuracy and cost-effectiveness of CCE. The study will include 973 participants who will undergo CCE and colonoscopy within the same day. This study will hopefully determine the true diagnostic accuracy of CCE compared with colonoscopy and, furthermore, determine the true cost-effectiveness of CCE [2]. ColoCap is set to begin recruiting patients from England, Scotland, and Wales in January 2025 [2].

## 3. Developmental Session I—Hardware

For the second session of the symposium, new developments for both CE and colonoscopy were presented, sparking discussions on ways to improve the existing hardware and addressing the constantly evolving needs of clinicians.

Two critical needs in the market were highlighted: oral drug delivery and gut sampling. The symposium’s keynote lecture focused on using robotics for therapeutic applications, introducing devices as small as 300 μm for drug delivery [20]. Microcontainers filled with antibiotics and coated with a PH-sensible film have proven efficient and could be the future for targeted drug delivery in the gut [21].

The importance of gut sampling was also discussed. Gut sampling seems to become essential due to the increasing evidence of the microbiome being linked not only to colorectal cancer but also to multiple diseases outside of the gut [22]. It seems possible that a proof-of-concept for a self-polymerizing system for gut sampling could be developed [23]. However, challenges arise due to the sample size limits and the need for instant preservation, which is still a developmental issue.

Alternatives to standard procedures, such as robotic colonoscopy and a new magnetic flexible endoscope, were also introduced. These procedures aim to reduce patient discomfort and pain by guiding the endoscope with outside magnetic force instead of pushing it with hand force [24].

Lastly, this session presented sonographic capsules capable of visualizing the subsurface of the mucosa [25]. However, a significant hurdle with this product remains the long developmental timeframe. This was further discussed, as the gap between clinicians and engineers widened. Unfortunately, that means prolonged times for the idea to develop and for the developers to produce [26].

Many of these developmental presentations were still far from the clinical practices around the world. However, the exchange of ideas and knowledge within a forum of experts is essential for future developments. The main perspective was, throughout all presentations, the possibility of providing better and more accurate, minimally invasive diagnostic modalities—while keeping the patient the main priority.

## 4. Developmental Session II—Software and Artificial Intelligence

The third and last session of day one introduced the recent advances in software and AI. There are still many individual aspects of capsule endoscopy that can benefit from AI algorithms. For instance, a large European study group named AICE (AI in CE) is one of the groups working towards developing multiple AI algorithms surrounding CCE [27]. They presented promising results of algorithms designed to assess cleansing and completeness, polyp matching, detection and segmentation, size estimation, and characterization. However, further evaluation and testing of the algorithms are still needed.

One of the main advantages of AI integration in capsule endoscopy is to address reader fatigue, a recurring topic in the discussions. We know, mostly from small bowel capsule research, that CE reader’s accuracy declines after the first study. This is also a well-known fact among endoscopists, whereby the completion rate and adenoma detection rate is known to decline drastically with successive procedures [28,29]. For CCE, this is related to experienced and novice readers but is particularly worse among experienced readers [30]. Furthermore, multiple studies indicate that physicians’ performance is disappointing regarding capsule reading due to inaccuracy and, primarily, due to a lack of consistency [31]. This area needs improvement if CCE will succeed in clinical practice, and therefore, it is an apparent place to introduce AI.

AI used in PCE will be the future in this field, because it can evaluate the entire GI tract without manual use. However, a valuable point was made about using explainable AI (XAI), as AI has been significantly associated with black-box medicine [32]. Therefore, this highlights the need for this process to be performed on data, which should be available across platforms and taught on multiple datasets, including different hardware and software, making the algorithms compliable to various systems and excluding monopolies in the field. It is of the utmost importance for the implementation of AI healthcare that traceability and patient data protection are the main focus during development [33,34]. The AICE group have, therefore, also allocated a large portion of their funding to ethical investigations within AI in CE [27].

AI offers a solution by providing consistent, objective assessments, even when clinicians may not always concur with the AI’s findings. Consistency remains a key advantage, ensuring reliable outcomes over time.

## 5. Clinical Session II—Commercial Solutions

In the first session of the second day, the industry representatives had the chance to present their recent developments and engage in discussions with the expert scientists and clinicians—once more, emphasizing the need for constant collaboration to shorten the gap that tends to widen between brilliant ideas and the time and costs for the development of the finished products.

This session also included some exciting findings from the CAPTURE EU project. They presented some of their most recent findings regarding the use of CCE in the European Union (EU) [35]. They found that CCE practices vary significantly throughout Europe, and critical factors influence the uptake of certain practices [35]. Some significant findings are that only 7% of the responses reported that CCE was included in the national guidelines, and only 45% had local services available [35]. The highlighted problems need to be assessed to widen the use and integration of CCE.

## 6. Clinical Session III—CCE Becoming a Regular Business

The last symposium (2023) focused on the remaining challenges in colon capsule endoscopy, including quality assurance, the cost-effectiveness of CCE, carbon footprint, and ethical aspects [15].

The discussions this year covered the current state of CCE services and recent innovations in CCE delivery. Research has shown that CCE is feasible in an out-of-clinic setting [36,37,38]. The latest study even suggests that CCE can be conducted entirely without contact with the clinic [37]. This study also highlighted patient perspectives, showing high satisfaction with the overall procedure, with 83.3% of participants indicating they would prefer to undergo CCE again at home [37]. Ongoing trials are further evaluating home delivery implementations, and it would be beneficial for more studies to evaluate different ways of implementing CCE into different healthcare systems.

One study on the acceptability of alternative colorectal cancer screening technologies was introduced, revealing no significant patient preference between CCE and traditional colonoscopy [39]. This underlines the need for broader, multinational studies with adaptable master protocols that can be adjusted to changes and findings throughout the research process. With large-scale studies like this, it would be possible to incorporate CCE alongside the already existing modalities and evaluate these on multiple healthcare platforms, while simultaneously comparing the data for best practice guidelines.

This session also included valuable insights from the nurse community. Emphasizing the need for acceptance among the clinical community when building any clinical service. Building a CCE service needs a clear vision and collaboration to achieve acceptance.

An English feasibility study, 5G-SUCCEEDS, is the first to introduce a remote CCE service and has found the home-delivered CCE to be a feasible and acceptable alternative to traditional hospital-based screening methods [40]. This study also included an analysis of carbon footprint. It has long been known that healthcare activities significantly contribute to the emission of greenhouse gases and waste production, and GI endoscopy is among the most significant contributors to the healthcare systems [41]. CCE has been thought to have a more negligible emission impact, mainly due to the amount of plastic waste associated with colonoscopy. However, a conference abstract reported an investigation of the environmental effects of colonoscopy and CCE [42]. They found that generated CO_2_e was 55.46 kg for colonoscopy and 48.21 kg for CCE, respectively, indicating far less improvement of CO_2_ emissions than originally thought [42]. Other studies have found that the carbon footprint of capsule endoscopy is mainly associated with the patient’s travels, and that the capsule and its components have a relatively low environmental impact, further indicating the great possibilities within home-delivered CCE systems [42,43]. As of right now, the previously thought climate advantage of CCE seems to be equal to colonoscopy. This indicates further challenges that must be solved for the full and acceptable implementation of CCE in a clinical setting.

It is important to highlight that the available data on carbon emission within CCE are still very limited. Even though the studies so far indicate reduced improvement of emissions, the final numbers are still to be evaluated.

Building a dedicated research platform could be crucial to advancing CCE research. This platform and studies like ColoCap would provide essential infrastructure for future research initiatives, which is highly needed in order to find the best way of implementing CCE in clinical practice.

## 7. Summary

To sum up the knowledge exchanged during the symposium in October 2024, we must acknowledge the significant improvements made to minimally invasive GI diagnostics throughout the last decade. It is of great advantage for patients around the world, and we can conclude that we are far from finished. The development needs to continue and stay up to speed with the newest technology.

CCE has been proven to be a valid and effective tool. However, the best use of the tool and the most effective delivery systems are still to be determined.

Studies on the gut microbiome and its association with health, in general, is an interesting topic, with lots of unturned stones waiting to be investigated.

Lastly, the importance of substantial collaborative interdisciplinary work between clinicians, engineers, and industry representatives must be acknowledged in order to achieve great success—and this is why multidisciplinary symposiums and international collaborations are and will continue to bring the highest benefit to GI diagnostics future.
